# Functional and Quality Profile Evaluation of Butters, Spreadable Fats, and Shortenings Available from Czech Market

**DOI:** 10.3390/foods11213437

**Published:** 2022-10-29

**Authors:** Barbora Lapčíková, Lubomír Lapčík, Tomáš Valenta, Tereza Kučerová

**Affiliations:** Faculty of Technology, Department of Food Technology, Tomas Bata University in Zlín, Nám. T. G. Masaryka 5555, 760 01 Zlin, Czech Republic

**Keywords:** butters, spreadable fats, shortenings, texture profile analysis, free fatty acids, differential scanning calorimetry, rheology, fluorescence spectrometry

## Abstract

The aim of this study was to assess the functional properties of butters, spreadable fats, and shortenings, collected from the Czech market, in correlation with their nutritional values declared by the producers. Various methods were applied to determine relevant parameters of the products. Using penetration tests, samples were characterized by specific textural attributes according to their composition and processing type, particularly for the presence of milk/vegetable fats. Using differential scanning calorimetry (DSC), thermal peaks corresponding to medium- and high-melting triacylglycerol fractions were detected in the ranges 15–16 °C and 31.5–34.5 °C, respectively. Rheological analysis revealed that the viscoelasticity of samples was related to frequency behavior of the fat structure, characterized by the dominance of elastic modulus (G′) over viscous modulus (G″) up to the frequency of 10 Hz. This indicated good emulsion stability of the products in the region of linear viscoelasticity. For spreadable fats, the structure was resistant to phase separation in the whole frequency range under study (0.1–100 Hz). The results showed that the applied techniques can be successfully used to characterize the processing and compositional quality of butters and vegetable fats.

## 1. Introduction

Butters and dairy alternatives, such as spreadable fats and shortenings, are involved in many food applications. Milk fat is a main component of butter products. Over a wide range of temperatures, milk fat is a mixture of solid crystals connected in a network and embedded in a liquid phase. Structural and compositional characteristics of these crystals evolve as function of temperature and time. Crystallization properties and polymorphism of milk fat in butter and vegetable fats in alternative products can be affected by many factors, e.g., sample dispersion state, cooling rate, shear applied during the processing, presence of minor lipid compounds (free fatty acids, phospholipids, mono- and diacylglycerols), and triacylglycerols (TAG) composition [1].

Milk (cream) from different sources can be utilized for the formation of butters and related products. As a low-cost and efficient alternative to butters, spreadable fats and shortenings are produced. They are characterized by specific mixtures of vegetable fats/oils and their processing. Currently, spreadable fats are produced by transesterification of triacylglycerols. Shortenings are prepared by controlled crystallization of fat substance, whereas the dispersion of TAG crystals is formed (ideally in the form of spatial network), and the dispersion medium is created by liquid triacylglycerols [2].

Crystallization of milk fat globules during aging of cream plays an important role in butter’s functional properties. The number and size of crystals in cream can be adjusted by controlling the temperature and time of the aging process and by temperature fluctuations before butter churning. Rapid cooling can cause a large quantity of small crystals in the cream, which can produce a firm butter, whereas slow cooling for a long time may yield a soft butter [3,4,5].

A convenient and fast technique, which enables determining the composition and crystallization profile of butters and vegetable fats, is differential scanning calorimetry (DSC), facilitating the analysis of various food components. DSC is neither expensive nor labor-intensive, and it can be applied in many kinds of food quality evaluations, including assessment of milk fat adulteration by vegetable oils or milk fat fraction characterization. DSC studies of milk and vegetable fat melting and crystallization involve the measuring of released or absorbed heat (normalized in J/g) at relevant heating/cooling rates. The principle of analysis lies in the fact that every fat has a unique composition of fatty acids and triacylglycerols, which are manifested in characteristic melting and crystallization curves [6,7,8].

The thermal behavior of milk fat, like all fats, is a direct reflection of the storage conditions under which the fat has been crystallized. It is relatively easy to set up a metastable crystalline state (β′ modification) in milk fat, which persists until the temperature is raised to the melting point of the metastable form. Repeated temperature cycling of butter products from refrigerated to room conditions allows the conversion of a metastable state to a more stable form (β), being adversely affected in the process of spreadability [9]. For spreadable fats, small β′ crystals are favored since they enable the formation of a good crystal network throughout the continuous lipid phase which is related to desirable sensory and textural properties [10].

Texture is one of the significant quality attributes of edible fats. It is based on the fat’s TAG profile and fatty-acid content [11]. Textural parameters are associated with the spreadability, appearance, and consumer acceptability of the products. Butter texture can be affected by different processing methods and treatments. It is attributed to the three-dimensional network of fat crystals in a continuous oil phase. The extent of crystallization and the ratio of solid to liquid fat are the primary determinants of butter consistency and other textural parameters [4,12].

In this study, the effect of processing type and composition on functional properties of butters and vegetable fats was examined. In particular, we focused on textural, rheological, crystallization, and melting properties, investigated using a combination of suitable techniques. The aim of this study was to access the properties and quality of market butters, spreadable fats, and shortenings from various functional aspects, and to present validation and product characterization. The applied methods were simultaneously utilized to evaluate the relevant parameters of butters and butter alternatives to determine the correlation between the data declared by the producers and the functional properties. On the basis of the results, characteristic parameters of the samples could be derived and used to generalize the functional profile of investigated products.

## 2. Materials and Methods

### 2.1. Materials

Different commercial brands of butters, spreadable fats, and shortenings, collected from the Czech market in the year 2018, were investigated in our study. The products were selected on a random basis to provide the highest variability of processing type and composition as possible. Samples were produced in Czech Republic, except for Rokitnianka butter from Poland. They included five types of butters (No. 1, No. 2, No. 3, No. 4, and No. 5), three types of spreadable fats (No. 6, No. 7, and No. 8), and two types of shortenings (No. 9 and No. 10). Three batches of each sample were tested. Before the analyses, samples were stored at refrigerated temperatures standardly used in the distribution chain (4 ± 1 °C) for 1 week. In Table 1, the products are characterized, including their producers, energy intake, and nutritional values.

All chemicals used in this study were purchased from certified companies and were of analytical grade. Cyclohexane (≥99.8%) was delivered by Sigma-Aldrich (USA), diethyl ether (≥99.8%) was delivered by Chromservis s.r.o. (Czech Republic), and ethanol absolute (≥99.8%) was delivered by Penta Chemicals Unlimited (Czech Republic). Potassium hydroxide and phenolphthalein indicator were obtained from Lachner, s.r.o. (Czech Republic).

### 2.2. Texture Profile Analysis

**Figure 1:**
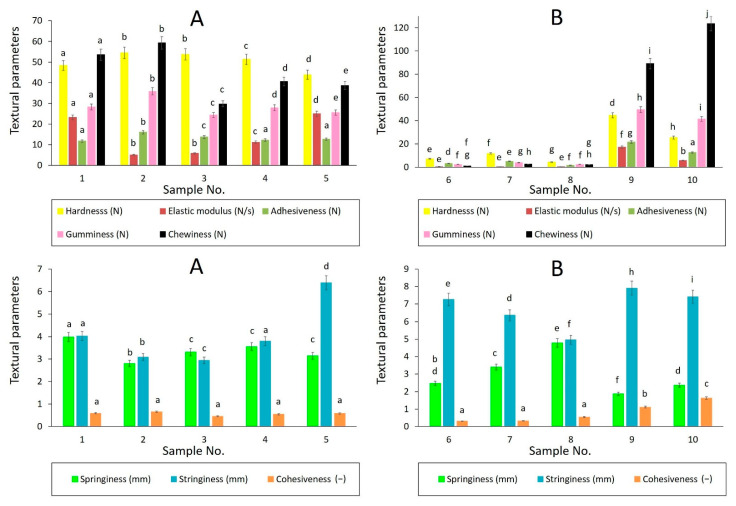
Texture profile analysis of butters (**A**), spreadable fats, and shortenings (**B**) using 5 mm distance penetration test (bar chart with 5% error bars). Textural parameters are described in the legend with the relevant units in the brackets; adhesiveness is expressed in absolute values. Different superscript letters for the same textural parameter indicate statistically significant differences between the samples (α ≤ 0.05, Tukey test).

Texture profile analysis was performed using a TA.XT. plus Texture Analyzer (Stable Micro Systems, United Kingdom). With the help of a cutting cylinder, samples of standard size 15 × 35 mm (thickness × diameter) were prepared, as can be seen in Figure 1. An aluminum cylindrical probe SMS P/50 (of 50 mm diameter) was used for double compression of samples equilibrated at temperature 15 ± 0.5 °C. This temperature was used with respect to the fat triacylglycerol melting properties [13] to achieve optimal sample penetration. Two measurement modes (probe settings) were applied. In the first mode, samples were squeezed twice to 5 mm distance (sample penetration) using a trigger force of 10× g. In the second mode, samples were compressed to 10% of their initial height (10% strain) using a trigger force of 5× g. In both modes, the probe test speed was 0.5 mm/s, with pre-test and post-test speeds of 1 and 10 mm/s, respectively.

The force and displacement of the probe were recorded as a function of time. The samples’ textural parameters were determined. Hardness (firmness) (*N*) represented the maximum force that occurred during a compression cycle, and the Young elastic modulus (*N*/s) was the modulus of sample deformability calculated as a gradient. Springiness (elasticity) (mm) was the height which sample recovered after its deformation between the end of the first and beginning of the second compression. Adhesiveness (*N*) was the maximum negative force required to pull the probe away from sample, i.e., to overcome attractive forces between the probe and sample surface. Cohesiveness (-) was related to the strength of sample internal bonds, and it was calculated as the ratio of area under force–time curve after the second compression to the area after the first compression. In addition to these primary textural characteristics, secondary parameters were determined. Stringiness (mm) was the distance to which the sample was extended during decompression before separating from the probe. Gumminess (*N*) represented the energy needed to disintegrate a semisolid sample to a state ready to be swallowed. Gumminess was calculated using the following equation:

(1) $$Gumminess=\frac{A_{2}}{A_{1}}\times F_{1},$$

where *A*_2_ is the area under the force–time curve after the second compression (*n*.s), *A*_1_ is the area under the force–time curve after the first compression (*n*.s), and *F*_1_ is the peak force during the first compression (*n*).

Chewiness (*N*) was considered as energy required to chew a solid sample to a state ready to be swallowed. This parameter was determined using the following equation:

(2) $$Chewiness=Gumminess\times \frac{L_{2}}{L_{1}},$$

where *L*_2_ is the distance from the beginning of the second compression to the peak force of the second compression (*L*_2_ is equal to the springiness) (mm), and *L*_1_ is the distance from the beginning of the first compression to the peak force of this compression (mm) [14,15,16].

### 2.3. Free Fatty Acids and Acid Number

The free fatty acid index and acid number of butters, spreadable fats, and shortenings were determined using the titration method. Briefly, 5 ± 0.5 g of fat sample was melted at 30–40 °C, and then mixed with an appropriate amount (10 mL) of pre-neutralized organic solvent (composed of 99.8% diethyl ether and absolute ethanol in the ratio 1:1). Under a continuous mild heating in a water bath, the solution was titrated using a potassium hydroxide alcoholic solution of standardized concentration 0.1 mol/L. Phenolphthalein alcoholic solution (1 wt.%) was used as an indicator. The titrant volume measured during sample titration was subtracted from the titration volume of the blank sample (without the fat).

Free fatty acid content (wt.%) was calculated using the following equation:

(3) $$Free\;fatty\;acids\;\left(FFA\right)\;index=\frac{V\times c\times M}{10\times m},$$

where *V* is the titrant volume during sample titration reduced by titrant volume during blank sample titration (ml), *c* is the concentration of potassium hydroxide solution (mol/L), *M* is the molar weight of oleic acid (282.47 g/mol) used as a representative fatty acid, and *m* is the weight of sample (g).

Acid number is the measure of free fatty acids amount in the fat and is expressed as the weight of potassium hydroxide (mg) needed to neutralize the acids (represented by oleic acid) contained in 1 g of fat. The following formula was used to calculate the acid number (mg/g):

(4) $$Acid\;number=\frac{56.11\times V\times c}{m},$$

where *V* is the titrant volume during sample titration reduced by titrant volume during blank sample titration (mL), *c* is the concentration of potassium hydroxide solution (mol/L), and *m* the weight of sample (g).

### 2.4. DSC Analysis

Differential scanning calorimetry (DSC) was performed on a DSC 1 Star System (Mettler-Toledo, Greifensee, Switzerland). The device was calibrated using an indium standard for temperature correction. Approximately 15 ± 1 mg of sample was inserted into an aluminum pan (ME-27331, 40 μL pan with pin) and sealed hermetically. An empty sealed pan was used as a reference. All samples were measured under the following temperature regime: dynamic heating from +25 °C to +70 °C at a heating rate of 10 °C/min, and holding at this temperature for 5 min to ensure that all nuclei of crystals were eliminated. Subsequently, cooling was applied from +70 °C to −40 °C at a cooling rate of 5 °C/min. Then, isothermal cooling was held at −40 °C for 3 min, followed by final heating to +70 °C (at heating rate 5 °C/min). Experiments were realized under nitrogen atmosphere at a flow rate of 50 mL/min. Thermally induced structural transitions (thermal events) were characterized by T_o_ (onset), T_p_ (peak), and T_e_ (endset) temperatures on DSC curves. Melting and crystallization peak temperatures (°C) were evaluated, and corresponding enthalpies (J/g) were determined according to the area under DSC curve [1,17,18,19,20,21].

### 2.5. Rheology

Rheological analysis was performed on a rheometer HAAKE RheoStress 1 (Thermo Fisher Scientific, Waltham, MA, USA). Plate–plate geometry (plate P35TiL, measuring plate cover MPC35) was applied with a measuring gap (i.e., distance between the plates) of 2 mm. The following parameters were observed: elastic storage modulus G′ (Pa), loss viscous modulus G″ (Pa), complex viscosity η* (Pa·s), and phase loss angle (tan δ = G″/G′). Measurements were carried out at a frequency of 0.1–10 Hz, pressure of 20 Pa, and temperature of 15 ± 0.5 °C. Temperature conditions were ensured by a circulating water bath Thermostat HAAKE AC 200 (Thermo Fisher Scientific, Waltham, MA, USA). Using HAAKE RheoWin software (Thermo Fisher Scientific, Waltham, MA, USA), sample dependencies of moduli and viscosity on frequency were evaluated.

### 2.6. Fluorescence Spectrometry

Fluorescence spectrometry of butters and vegetable fats was realized using a Shimadzu RF-1501 spectrophotometer (Shimadzu, Kyoto, Japan). The excitation wavelength was 220–360 nm, and the emission wavelength was 300–800 nm. Fluorescence intensity was read automatically in arbitrary units (AU) at specific wavelengths (nm) of excitation/emission spectra. Quartz cuvettes 6030-UV Herasil (Hellma Analytics GmbH & Co. KG, Müllheim, Germany) of 10 mm optical length adjusted for the UV/Vis region were used for the analyses.

Samples of butters, spreadable fats, and shortenings were melted at a temperature between 30 and 40 °C. Liquid samples were homogenized by addition of nonpolar solvent (99.9% cyclohexane). Concentrations of solutions were adjusted to detect optimal fluorescence intensity dependent on the wavelength: for butters, 1% and 2% (*w*/*v*) solutions, i.e., 1 g/100 mL and 2 g/100 mL, respectively; for spreadable fats and shortenings, 10% and 50% (*w*/*v*) solutions.

### 2.7. Statistical Analysis

Data were analyzed using one-way analysis of variance (ANOVA method). Differences in the mean values among statistical groups were tested at a significance level of α ≤ 0.05. The Tukey test was applied for multiple comparisons (statistical ranking) of the mean responses to treatment groups (α ≤ 0.05) to evaluate if they are greater than would be expected by chance. Statistical software SigmaStat version 2.03 (Systat Software, Inc., Chicago, IL, USA) was used for data testing. All experiments were performed in at least three replicates.

## 3. Results

### 3.1. Texture Profile Analysis

**Figure 2:**
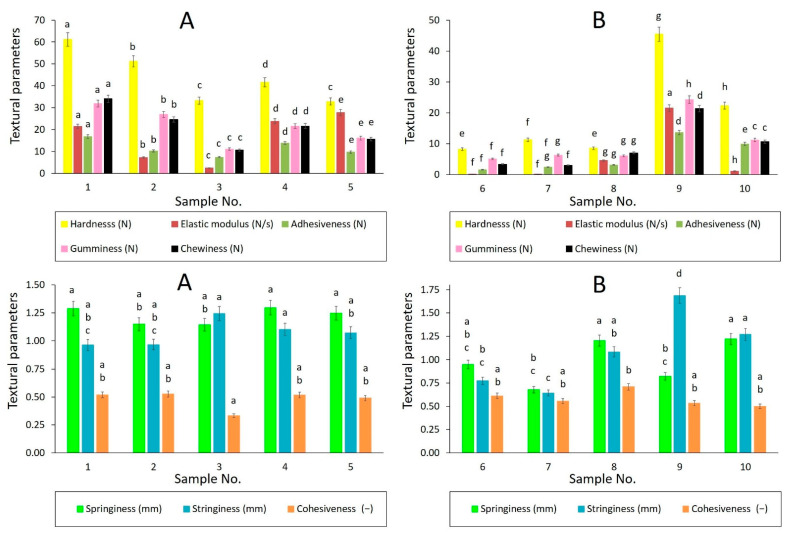
Texture profile analysis of butters (**A**), spreadable fats and shortenings (**B**) using 10% strain penetration test (bar chart with 5% error bars). Textural parameters are described in the legend with the relevant units in the brackets; adhesiveness is expressed in absolute values. Different superscript letters for the same textural parameter indicate statistically significant differences between the samples (α ≤ 0.05, Tukey test).

As presented in Figure 1 and Figure 2, similar textural behavior was determined for samples of the same product type (butters, spreadable fats, and shortenings) applying different methods (5 mm distance, 10% strain). Accordingly, the same generalizations can be made for butters, as well as spreadable fats. Investigated butters showed relatively high values of hardness, elastic modulus, gumminess, and chewiness, in comparison to spreadable fats. Relatively large differences within the group of butters were found for the Young elastic moduli. These findings are consistent with the observations of Espert et al. [22], who characterized butters of higher hardness compared to spreadable fats but lower compared to anhydrous milk fats. Lower spreadability and adhesiveness of butter products, prepared from ordinary cow milk, can be associated with their fatty-acid composition, characterized by higher long-chain saturated fatty-acid content [23].

Remarkable findings were observed for shortenings No. 9 and No. 10 which corresponded to butters with several textural attributes (relatively high values of chewiness, gumminess, Young elastic modulus, and hardness). This fact can be related to the nutritional characteristics of the shortenings, as presented in Table 1; the shortenings had relatively high total fat content, compared to spreadable fat products. In particular, the relatively high content of saturated fatty acids in the shortenings could lead to a more rigid structure. Moreover, shortening No. 10 contained 15% milk fat, and shortening No. 9 involved dried whey powder in the recipe, which may have influenced their texture profiles. In the case of shortening No. 10, relatively lower values of hardness compared to butters and another shortening could be explained by the presence of spring milk fat, which tends to be softer than summer milk fat [20]. This fact can also be confirmed by the DSC analysis results, which revealed the presence of low-melting TAG fractions in sample No. 10, typical for spring milk fat, as discussed below (see Section 3.3).

In contrast to butters and shortenings, samples No. 6, No. 7, and No. 8 behaved as typical spreadable fats with relatively low hardness, gumminess, and chewiness. This may be attributed to relatively low total fat content and/or emulsifier present in the fats, affecting their crystal network structure and emulsion stability [22,24].

### 3.2. Free Fatty Acids and Acid Number

**Figure 3:**
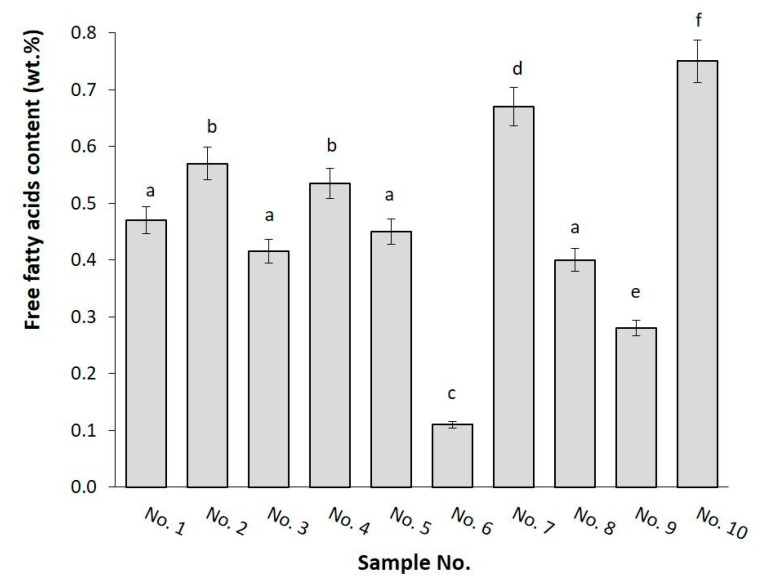
Free fatty acids (wt.%) in butters, spreadable fats, and shortenings (bar chart with 5% error bars). Different superscript letters above the bars indicate statistically significant differences between the samples (α ≤ 0.05, Tukey test).

The free fatty acid (FFA) index (wt.%) is an essential parameter used to control the quality of dairy products. We applied this index to assess the quality of butters and compare it with vegetable fats. The FFA index represents the percentage of free fatty acids, such as oleic, myristic, palmitic, and other acids involved in the product. Values of the FFA index (wt.%) are presented in Figure 3. The relations among the samples correspond to the acid number expressed in mg of potassium hydroxide per 1 g of fat. Using one-way analysis of variance (ANOVA), the samples were considered as one statistical set which differed significantly (at significance level α ≤ 0.05).

As evident from the graph (Figure 3), the free fatty-acid content of all samples was below the limit of 1 wt.% FFA (2 mg/g). At optimal conditions, FFA should be close to zero. The relatively high FFA content in shortening No. 10 (0.77 wt.%, 1.53 mg/g) could be explained by the various storage conditions within the supply chain [25]. In addition to 85% vegetable fats, shortening No. 10 contained 15% milk fat, as stated in Table 1. On the other hand, spreadable fat No. 6 exhibited the lowest FFA content (0.215 mg/g) among all samples. This can be explained by the relatively low total fat content (39 wt.%) in the product. However, no general relationship was found between the amount of free fatty acids and total fat content in the examined group of market products.

### 3.3. DSC Analysis

On the basis of DSC curves, fat crystallization and melting peaks were analyzed, as summarized in Table 2. Thermograms are presented in Figures S1–S10 of Supplementary Materials. For cooling cycle of butters, the first crystallization peak related to medium melting fractions was observed in a temperature range of 15.0 ± 1.5 °C. The second crystallization peak associated with low melting fractions was determined at 9.0 ± 0.7 °C, dependent on the sample examined [17] (the peaks are not stated in the table). Within the crystallization process of water, a sharp exothermic peak was detected in the temperature range from −9 to −20 °C due to the water supercooling phenomenon forming stable nuclei of crystal size [9]. Detected values were in relation to the content and character of water present in the emulsion system and varied from one sample to another, because nucleation is a stochastic event [26].

During reheating, the first endothermic peak related to ice melting was observed around the temperature of 0 °C, depending on the salt content in the products. For butters, the ice melting peak fluctuated in a narrow range (from 0.02 to 0.37 °C), due to a low amount of salt (Table 1). The two endothermic peaks were associated with melting of milk fat polymorphs. The first peak was detected at 15.1 ± 0.5 °C for Czech butter products, and at 16.1 °C for No. 1 butter from Poland, as presented in Table 2. This peak can be related to triacylglycerol medium-melting fractions, when a polymorphic transition from the α- to metastable β′-form of milk fat occurs [17]. The second endothermic peak at 31.5–34.5 °C can be attributed to high-melting fractions, characterized by longer hydrocarbon chains [7], i.e., to the melting process of the most stable milk fat crystallization form (β) [9]. Above this temperature, the liquid phase was formed.

As presented in Table 2, relevant thermograms were also detected for spreadable fats (No. 6, No. 7, and No. 8). For spreadable fats and shortenings, the variations in ice melting peak temperature were higher (from −0.12 to +0.83 °C), compared to butters. The process of ice melting was probably influenced by the emulsifiers present in the vegetable fats and the processing technology. In comparison to butters, water in spreadable fats crystallized in a relatively narrow temperature range of approximately −14 to −17 °C, which could indicate a good dispersion of water phase in the oil phase (i.e., stable systems with reduced phase separation). It can be expected that the ratio between vegetable fats and oils in these products (such as palm oil, sunflower oil, coconut fat, Shea butter, etc.) affects their crystallization properties. Beyond the water crystallization peak, small exothermic peaks in the temperature range of −20.5 to −30.5 °C were detected (not shown in the table). These peaks of minor enthalpy change can be related to the crystallization of vegetable oils, and their parameters were dependent on the unsaturated fatty-acid content in investigated products. For the shortenings (No. 9, No. 10), small crystallization peaks in the same temperature range were observed.

In the reheating cycle, several melting fractions of TAGs were indicated for spreadable fats. Detected endothermic peaks corresponded to the occurrence of saturated and unsaturated fatty acids present in transesterified fats of the products. Peaks recoded at relatively high temperatures (between approximately 44 and 51 °C) could be explained by the content of TAGs higher melting fractions [20].

In the case of shortenings, a melting peak at 8.5 °C was recorded for sample No. 10. This peak can be attributed to the presence of low-melting milk fat fractions; there was 15% milk fat declared by the producer, which might have come from spring milk, containing more low-melting TAG fractions [20]. For both shortenings (No. 9 and No. 10), an endothermic peak at temperature around 37 °C was observed, associated with higher melting fractions of TAGs.

### 3.4. Rheological Behavior

Relations of viscoelastic moduli and complex viscosity with frequency were evaluated in order to describe rheological behavior of butters, spreadable fats, and shortenings. Because edible fats are dynamically evolving systems, temperature and storage conditions have a significant effect on the properties determined [27]. Using 15 ± 0.5 °C as the optimal measuring temperature, differences in rheological parameters of the samples could be distinguished [28].

Butters exhibited an order-of-magnitude higher complex viscosity (η*) and viscoelastic moduli than spreadable fats. However, rheological parameters of shortenings were relatively comparable with the values determined for butters, which can correlate with their composition and similar textural properties. On a logarithmic scale, the complex viscosity of all samples decreased linearly with increasing frequency, although a nonlinear frequency dependency of η* was observed for butters and shortenings in the frequency above 10 Hz, limiting their linear viscoelastic region (LVR) under study. The elastic component (G′) of all samples predominated over the viscous (G″) in the region between 0.1 and 10 Hz (tan δ < 1).

**Figure 4:**
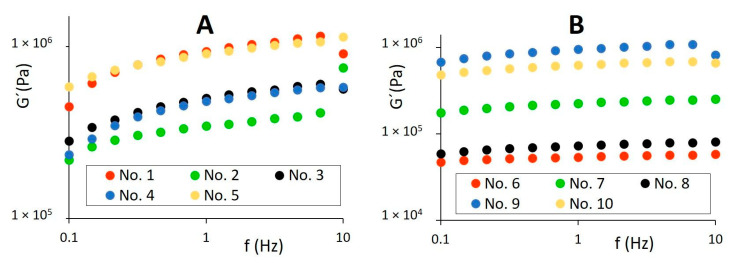
Elastic modulus (G′) of butters (**A**), spreadable fats, and shortenings (**B**) dependent on frequency f in the region 0.1–10 Hz on logarithmic scale.

**Figure 5:**
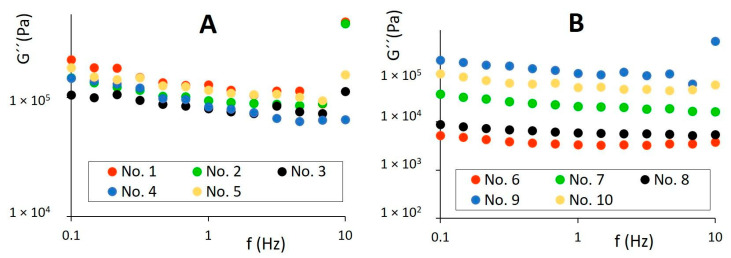
Viscous modulus (G″) of butters (**A**), spreadable fats, and shortenings (**B**) dependent on frequency f in the region 0.1–10 Hz on logarithmic scale.

**Figure 6:**
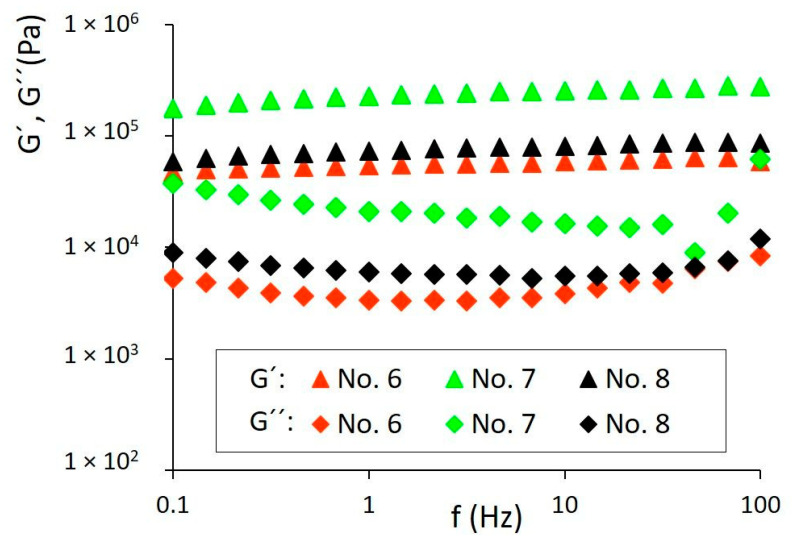
Elastic modulus G′ (represented by triangles) and viscous modulus G″ (represented by diamonds) for spreadable fats in the frequency range 0.1–100 Hz on logarithmic scale.

As can be seen in Figure 4 and Figure 5, the linear dependency of viscoelastic moduli was found for studied samples in LVR, indicating good emulsion stability between oil and water phases in the products. For spreadable fats, G′ and G″ moduli fluctuated in a relatively narrow range, and elastic character prevailed in the entire frequency region (0.1–100 Hz) applied (tan δ < 1), as illustrated in Figure 6. The ability of spreadable fats to store energy was relatively stable within the whole frequency range applied, forming a plateau region. The viscous character of samples was mildly decreasing at frequencies to 10 Hz; above this frequency, a slight increase in G″ values was observed. In comparison to other spreadable fats, sample No. 7 was characterized by a higher elastic and loss modulus, which correlated with higher hardness observed among the spreads by texture analysis (Figure 1 and Figure 2).

### 3.5. Fluorescence Spectrometry

Using fluorescence spectrometry, excitation/emission spectra were recorded, being typical for specific fats [29]. In the wavelength range 340–405 nm, emission peaks were detected for all butters under study, corresponding to saturated fatty acids (myristic, palmitic, and stearic) which are present in milk fats [30]. In addition to butters, a peak at 349 nm was found for shortening No. 10, which can be explained by the presence of 15% milk fat in the product, as stated in Table 1.

For butters, an emission peak around 370–375 nm, which can be related to vitamin D, and a peak around 390 nm, corresponding to vitamin K, were observed. For shortening No. 10, a peak around 480 nm was detected, which could be associated with the presence of vitamin A [31]. In the wavelength range of approximately 415–540 nm, emission peaks were observed for spreadable fats and shortenings. These peaks can be related to unsaturated fatty acids (oleic, linoleic, and α-linoleic acids), which are the main components of vegetable oils and fats. In particular, peaks detected in spreadable fats (No. 6, No. 7, and No. 8) at relatively low wavelengths (around 420 nm) could be associated with the presence of oleic acid; as reported by Croce et al. [32], this peak position ranged from about 415 nm to 430 nm, which is in excellent agreement with the results of our study.

Fluorescence spectra can be substantially influenced by the storage conditions; elevated temperature and sunlight exposition may initiate fat oxidation. The formation of oxidation products, such as aldehydes and ketones, can be appointed by emission peaks detected above 550 nm [31]. For all samples in our study, no emission peaks related to oxidation products were found, indicating proper handling and appropriate storage of investigated products.

## 4. Discussion

Values of textural parameters were related to sample composition and homogeneity, which play an important role in the texture and spreadability [33]. Butters were characterized by relatively high hardness, elastic modulus, gumminess, and chewiness, compared to spreadable fats. This could be explained by the higher ratio of saturated fatty acids which contributes to the hardness and poor spreadability of butters at refrigeration temperatures [34], and this was also demonstrated in our study at a temperature of 15 °C applied by texture analysis. However, statistically significant differences were found among many textural parameters determined for butter samples, which could be related to the differences in milk fatty-acid composition and/or processing conditions. As reported by Bobe et al. [34] and Ramaswamy et al. [35], the variations among the cow diets can provide butters with different textural properties. In particular, the hardness (stiffness) of butters is influenced by the fatty-acid composition; it can be expected that the unsaturated fatty acids inhibit the formation of crystals in the butter samples and, thus, the hardness of the samples can decrease due to a low number of solid triglycerides [36]. This may be the case for sample No. 5, which provided significantly lower values of hardness between the butter samples. Spreadable fats were characterized by substantially lower values of hardness, young elastic modulus, springiness, adhesiveness, gumminess, and chewiness in comparison to butters and shortenings. For other textural parameters, i.e., springiness, cohesiveness, and stringiness, no general relationship between the content and composition of total fat in the samples and the values of textural parameters was found.

The data of the FFA index indicate that investigated samples are in agreement with the standards generally imposed on this type of market products [37,38,39]. The compliance of the supply chain with the recommended storage conditions (i.e., maximum temperature, relative humidity, and direct sunlight prevention) [37,40] can be deduced from the results.

Using DSC analysis, crystallization and melting profiles of the samples were determined. The thermal behavior of the samples was based on their fatty-acid composition, particularly on the ratio of saturated and unsaturated fatty acids, which can be influenced by many factors during milk/vegetable fat production [1,17,18,19,20,21]. Notably, for spreadable fats and shortenings, the process of transesterification and triacylglycerol (TAG) fractionation of structural fat mixed with liquid oils [2] could have led to differences in crystallization and melting behavior among the studied samples. The number of carbons in the fatty-acid chains and the degree of unsaturation might be of great importance for the products [41]. The thermal behavior of butters was in relation to the presence of odd-numbered fatty acids which melt at lower temperatures compared to even-numbered fatty acids, and to conjugated linoleic acid in milk fat [42]. On the basis of this knowledge, thermal profiles of samples indicated compositional quality of the products, as proven by the detection of lower/higher triacylglycerol melting fractions.

Rheological results corresponded with other data determined by applied techniques. In the linear viscoelastic region (LVR), the elastic modulus G′ was higher than viscous modulus G″, showing typical behavior of a solid matrix compared to a liquid (phase angle tan δ < 1) [43], as determined for all samples under study. In LVR, the samples’ ability to store energy and maintain their structure was retained. At higher frequencies, the matrix of butters and shortenings was not able to absorb mechanical energy inserted, and a change in emulsion structure led to the phase separation [9,28,33]. The spreadable fats exhibited stable ability to store energy in the applied frequency range, forming a plateau region. It can be concluded that the spreadable fats exhibited high tolerance to phase separation in all frequencies applied. The data of our study are comparable with Espert et al. [22], who measured viscoelastic behavior of low-fat spreads in the same frequency range.

Fluorescence spectrometry was used for identification of chemical composition of the samples. The emission peaks found in the range from 415 to 540 nm confirmed the presence of unsaturated fatty acids typical for vegetable fats and oils for spreadable fats and shortenings. This technique was also applied to validate the presence of the oxidation products. No emission peaks above 550 nm wavelength were detected, which indicates zero presence of the oxidation compounds due to inadequate storage conditions. These findings were consistent with similar studies on the oxidative stability of butters and edible oils by Melo et al. [44], Mallia et al. [45], Veberg et al. [46], and Wold et al. [47], who also investigated the lipid oxidation products.

## 5. Conclusions

Various butters, spreadable fats, and shortenings were investigated in relation to their processing and composition in order to define differences and similarities in functional parameters of the products and in relation to the content and character of fat (unsaturated/saturated fatty acids). Through texture analysis, shortenings provided several attributes (chewiness, gumminess, Young elastic modulus, and hardness) which were more typical for butters than spreadable fats, and these could be ascribed to the relatively high content of fat and saturated fatty acids. DSC curves of butters showed two melting peaks at temperatures around 15–16 °C and 31.5–34.5 °C, related to polymorphic forms of triacylglycerol fractions. For spreadable fats and shortenings, various peaks were determined, dependent on their vegetable fats/oils composition. Due to the presence of vegetable fats, spreadable fats and shortenings provided comparable thermal behavior. Using rheological analysis, butters exhibited viscoelastic moduli of an order of magnitude higher than spreadable fats, as a function of the high content of milk fat and saturated fatty acids forming a relatively rigid butter structure. This is consistent with the fact that the hardness of butters was comparable with shortenings but significantly higher than the hardness of spreadable fats. For spreadable fats, a plateau region of elastic modulus was observed in the frequency range 0.1–100 Hz, whereas, for butters and shortenings, the linear viscoelastic region was limited to frequency of 10 Hz. To ensure the functional quality of butters and vegetable fats, compliance with the European standards plays a vital role. Simultaneous application of the methods used in this study can be an appropriate tool to assess quality and composition of butters and their alternatives available in the markets. The quality of spreadable fats as main alternatives could be enhanced by applying the data of our research in relation to possible alterations of product composition (e.g., total fat content, mixture of vegetable fats/oils, and vitamin supplementation), based on the market demands. From a functional and nutritional point of view, spreadable fats showed many advantageous properties compared to butters, such as lower hardness and adhesiveness and higher spreadability; furthermore, due to the lower total fat content, they can provide a lower energy intake for the consumers.

## Figures and Tables

**Table 1:** Nutritional characteristics of butters, spreadable fats, and shortenings per 100 g of the products.

**Nutritional** **Values/Energy** **Intake per 100 g**	**Products Name, Producer**				
	**Rokitnianka Butter (No. 1),** **Mleczarska** **Rokitnianka**	**Olomoucké Butter (No. 2),** **OLMA, a.s.**	**Tatra Farmářské Butter (No. 3),** **Mlékárna** **Hlinsko, a.s.**	**Česká Chuť Butter (No. 4),** **OLMA, a.s.**	**Dr. Halíř Butter** **(No. 5), Mlékárna Čejetičky, spol. s r.o.**
Energy intake (kJ/kcal)	3060/744	3095/739	3134/762	3095/739	3056/743
Total fat (g) ^a^ (of which saturated fatty acids)	82.1 (57.4)	83.0 (51.0)	84.0 (55.0)	83.0 (51.0)	82.0 (52.0)
Saccharides (g) (of which monosaccharides)	0.6 (0.6)	0.8 (0.8)	0.8 (0.8)	0.8 (0.8)	0.7 (0.7)
Proteins (g)	0.7	0.6	0.7	0.6	0.6
Salt (g)	0.02	0.02	0.10	0.02	0.02
Vitamin A (µg)	-	-	-	-	-
Vitamin D (µg)	-	-	-	-	-
Vitamin E (mg)	-	-	-	-	-
	**Perla** **(No. 6),** **UNILEVER ČR, spol. s r.o.**	**Rama** **Classic** **(No. 7),** **UNILEVER ČR, spol. s r.o.**	**Flora** **(No. 8),** **UNILEVER ČR, spol. s r.o.**	**Hera** **(No. 9),** **UNILEVER ČR, spol. s r.o.**	**Zlatá Haná** **(No. 10),** **OLMA, a.s.**
Energy intake (kJ/kcal)	1450/346	2241/535	1673/405	2666/637	2762/659
Total fat (g) ^a^ (of which saturated fatty acids)	39.0 (10.0)	60.0 (24.0)	45.0 ^b^ (10.0)	72.0 (34.0)	74.0 ^c^ (35.0)
Saccharides (g) (of which monosaccharides)	0 (0)	0.5 (0.5)	<0.5 (<0.5)	<0.5 (<0.5)	0.8 (0.8)
Proteins (g)	0	<0.5	<0.5	<0.5	0.6
Salt (g)	0.32	0.23	0.52	0.21	0.02
Vitamin A (µg)	800 (100%) ^d^	800 (100%) ^d^	800 (100%) ^d^	800 (100%) ^d^	-
Vitamin D (µg)	7.5 (150%) ^d^	7.5 (150%) ^d^	7.5 (150%) ^d^	7.5 (150%) ^d^	-
Vitamin E (mg)	18 (150%) ^d^	7.5 (60%) ^d^	14 (120%) ^d^	-	-

^a^ The values of total fat are related to milk fat in butters and vegetable fats in spreadable fats and shortenings. ^b^ For No. 8, the producer declared the presence of polyunsaturated fatty acids (PUFAs) in total fat, i.e., omega-3 and omega-6 essential fatty acids. ^c^ For No. 10, the producer declared 58% vegetable fats and 15% milk fat per 100 g of the product. ^d^ The percentages of vitamins are related to the reference daily energy intake for an average weight adult person (8400 kJ/2000 kcal). A hyphen indicates that no value was declared by the producer.

**Table 2:** DSC results of butters, spreadable fats and shortenings applying cooling process from +70 °C to −40 °C (at cooling rate 5 °C/min), and heating process from −40 °C to +70 °C (at heating rate 5 °C/min).

Sample	Crystallization Peak (Cooling)		1st Melting Peak (Heating)		2nd/3rd Melting Peak (Heating)	
	T_c_ (°C)	∆H (J/g)	T_p_ (°C)	∆H (J/g)	T_p_ (°C)	∆H (J/g)
No. 1	−14.09 ± 0.14 ^a^	32.22 ± 0.47 ^a^	16.15 ± 0.12 ^a^	18.13 ± 0.24 ^a^	34.20 ± 0.21 ^a^	192.41 ± 0.48 ^a^
No. 2	−19.66 ± 0.18 ^b^	34.21 ± 0.20 ^b^	15.10 ± 0.13 ^b^	30.60 ± 0.35 ^b^	31.55 ± 0.25 ^b^	66.79 ± 0.29 ^b^
No. 3	−16.92 ± 0.22 ^c^	30.61 ± 0.28 ^c^	15.09 ± 0.10 ^b^	34.35 ± 0.26 ^c^	31.24 ± 0.12 ^b^	40.59 ± 0.37 ^c^
No. 4	−13.32 ± 0.19 ^d^	38.87 ± 0.30 ^d^	15.34 ± 0.11 ^b^	14.21 ± 0.15 ^d^	31.84 ± 0.27 ^b,c^	38.54 ± 0.31 ^d^
No. 5	−9.16 ± 0.15 ^e^	35.20 ± 0.28 ^e^	15.49 ± 0.22 ^b^	14.54 ± 0.17 ^d^	34.38 ± 0.19 ^a^	148.99 ± 0.52 ^e^
No. 6	−15.83 ± 0.10 ^f^	42.61 ± 0.31 ^f^	-	-	- 44.57 ± 0.23 ^d^	- 335.97 ± 0.40 ^f^
No. 7	−16.91 ± 0.18 ^c^	74.34 ± 0.29 ^g^	-	-	32.30 ± 0.19 ^c^ 51.11 ± 0.17 ^e^	417.88 ± 0.55 ^g^ 8.50 ± 0.12 ^h^
No. 8	−14.46 ± 0.09 ^a^	42.90 ± 0.15 ^f^	24.57 ± 0.16 ^c^	83.69 ± 0.35 ^e^	- 50.83 ± 0.14 ^e^	- 57.29 ± 0.20 ^i^
No. 9	−16.71 ± 0.12 ^c^	58.72 ± 0.26 ^h^	-	-	37.57 ± 0.28 ^f^ 50.01 ± 0.19 ^g^	316.11 ± 0.48 ^j^ 26.56 ± 0.11 ^k^
No. 10	−21.60 ± 0.28 ^g^	39.01 ± 0.15 ^d^	8.48 ± 0.20 ^d^	22.18 ± 0.14 ^f^	34.12 ± 0.10 ^a^	43.35 ± 0.19 ^l^

T_c_—crystallization peak; ∆H—enthalpy change; T_p_—melting peak. Results of thermal parameters are stated as the arithmetic mean ± standard deviation of three means of sample batch triplicate measurements. The hyphen means that no peak in the corresponding temperature range was detected. Different superscript letters in the same column indicate statistically significant differences between the samples (α ≤ 0.05, Tukey test).

## Data Availability

The datasets generated in this study are available on request to the corresponding author.

## Supplementary Materials

The following supporting information can be downloaded at https://www.mdpi.com/article/10.3390/foods11213437/s1: Figure S1. DSC thermograms of detected crystallization peaks on cooling curve (blue color) and melting peaks on heating curve (yellow color) for sample No. 1; Figure S2. DSC thermograms of detected crystallization peaks on cooling curve (blue color) and melting peaks on heating curve (yellow color) for sample No. 2; Figure S3. DSC thermograms of detected crystallization peaks on cooling curve (blue color) and melting peaks on heating curve (yellow color) for sample No. 3; Figure S4. DSC thermograms of detected crystallization peaks on cooling curve (blue color) and melting peaks on heating curve (yellow color) for sample No. 4; Figure S5. DSC thermograms of detected crystallization peaks on cooling curve (blue color) and melting peaks on heating curve (yellow color) for sample No. 5; Figure S6. DSC thermograms of detected crystallization peaks on cooling curve (blue color) and melting peaks on heating curve (yellow color) for sample No. 6; Figure S7. DSC thermograms of detected crystallization peaks on cooling curve (blue color) and melting peaks on heating curve (yellow color) for sample No. 7; Figure S8. DSC thermograms of detected crystallization peaks on cooling curve (blue color) and melting peaks on heating curve (yellow color) for sample No. 8; Figure S9. DSC thermograms of detected crystallization peaks on cooling curve (blue color) and melting peaks on heating curve (yellow color) for sample No. 9; Figure S10. DSC thermograms of detected crystallization peaks on cooling curve (blue color) and melting peaks on heating curve (yellow color) for sample No. 10.
