# Supplementary material for: Functional and Quality Profile Evaluation of Butters, Spreadable Fats, and Shortenings Available from Czech Market
Source: Foods. 2022 Oct 29;11(21):3437. doi: 10.3390/foods11213437 (PMC9658663; doi:10.3390/foods11213437)
Supplement: Supplementary file 1 [file foods-11-03437-s001.zip › foods-1982386-supplementary.pdf]

Supplementary material

Functional and Quality Profile Evaluation of Butters, Spreadable Fats, and Shortenings Available from Czech Market

Barbora Lapčíková, Lubomír Lapčík *, Tomáš Valenta and Tereza Kučerová

Faculty of Technology, Department of Food Technology, Tomas Bata University in Zlín, nám. T. G. Masaryka 5555, 760 01 Zlin, Czech Republic
* Correspondence: lapcikl@seznam.cz; Tel.: +420-576-035-115

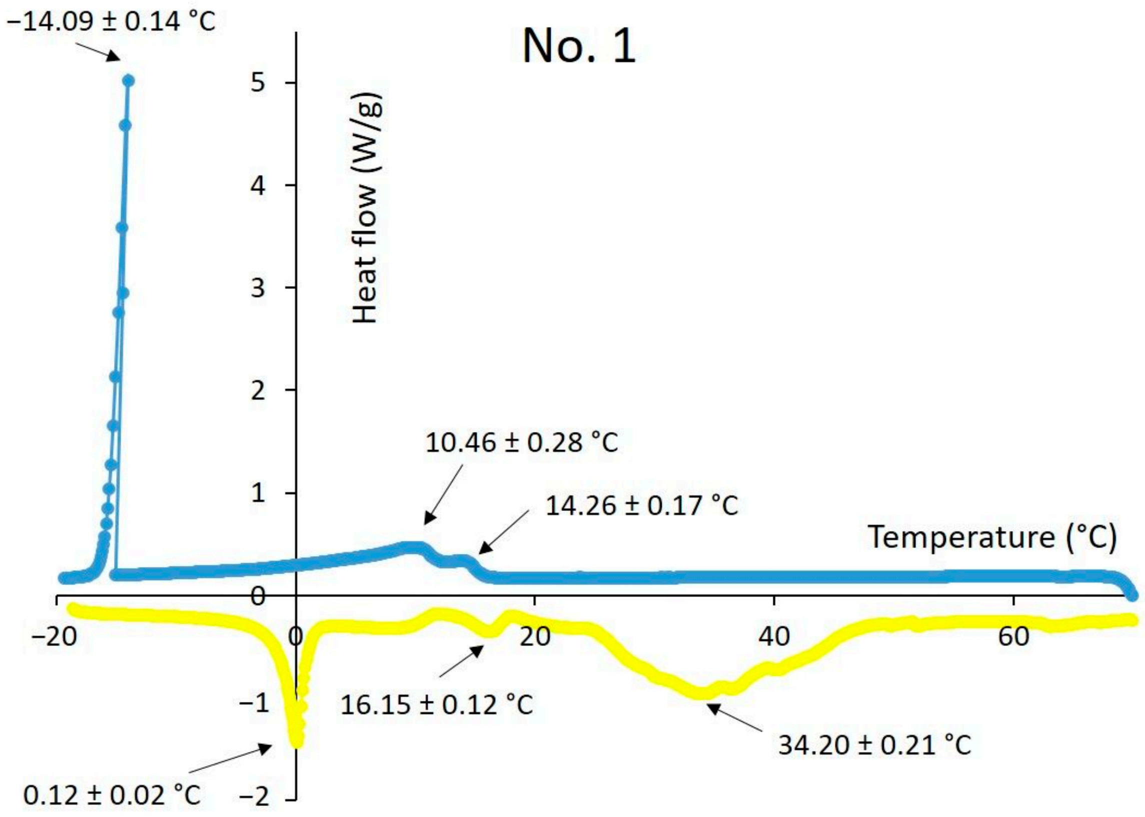

Figure S1: DSC thermograms of detected crystallization peaks on cooling curve (blue colour) and melting peaks on heating curve (yellow colour) for sample No. 1.

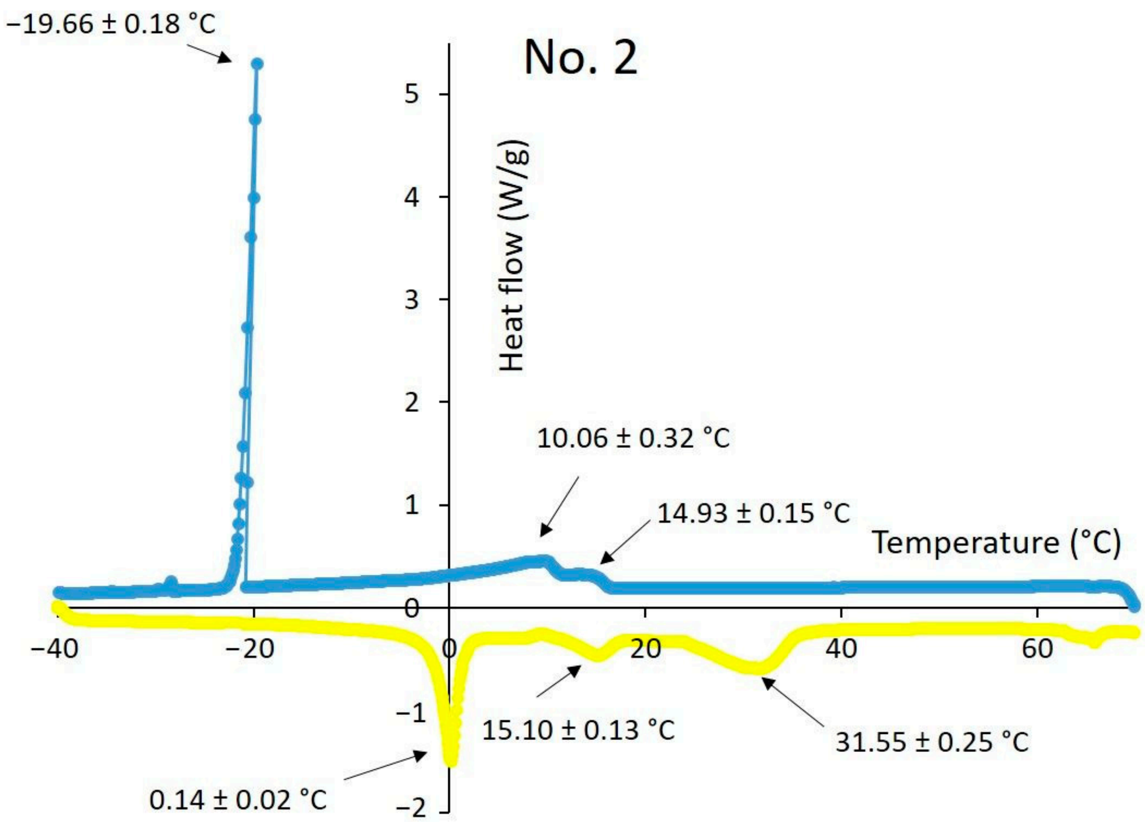

Figure S2: DSC thermograms of detected crystallization peaks on cooling curve (blue colour) and melting peaks on heating curve (yellow colour) for sample No. 2.

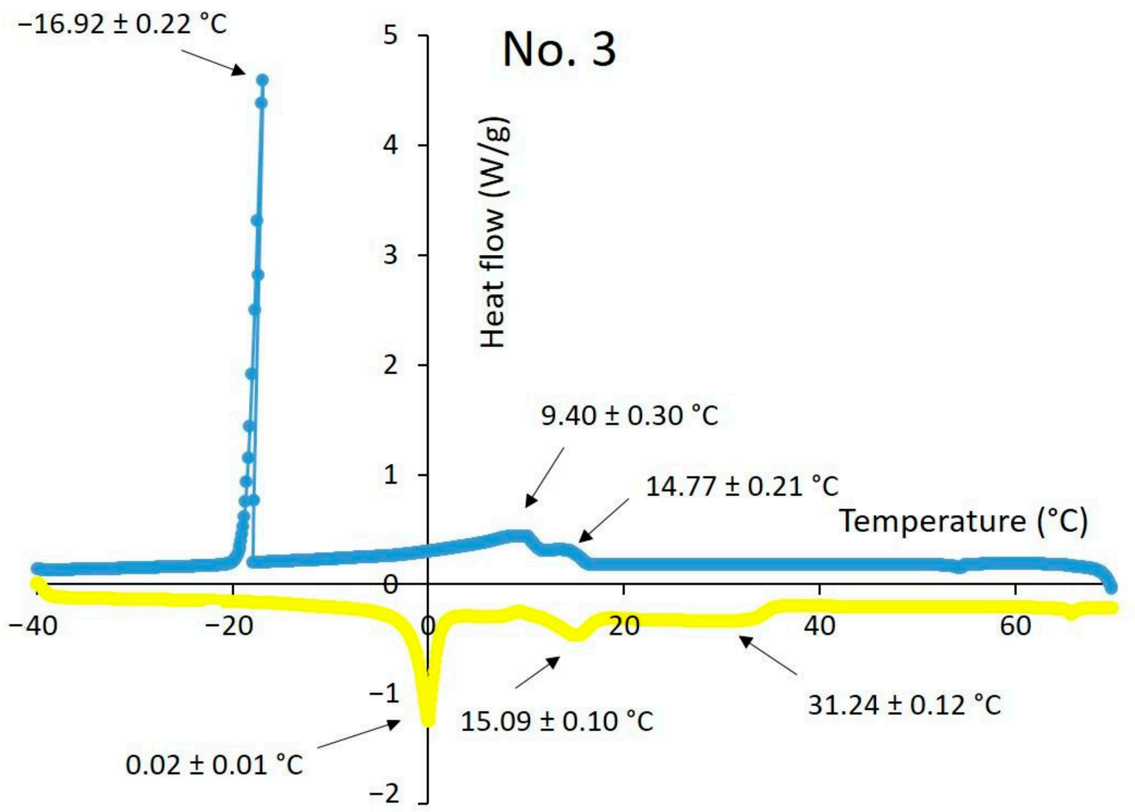

Figure S3: DSC thermograms of detected crystallization peaks on cooling curve (blue colour) and melting peaks on heating curve (yellow colour) for sample No. 3.

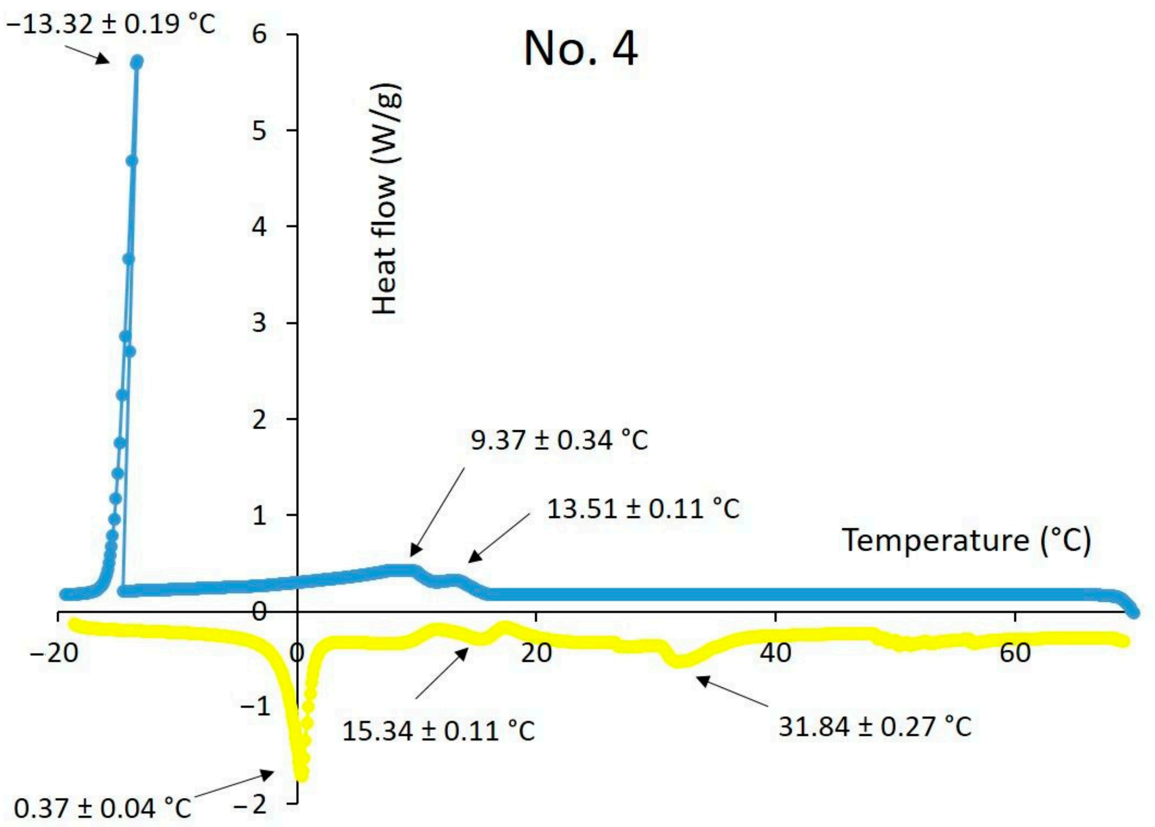

Figure S4: DSC thermograms of detected crystallization peaks on cooling curve (blue colour) and melting peaks on heating curve (yellow colour) for sample No. 4.

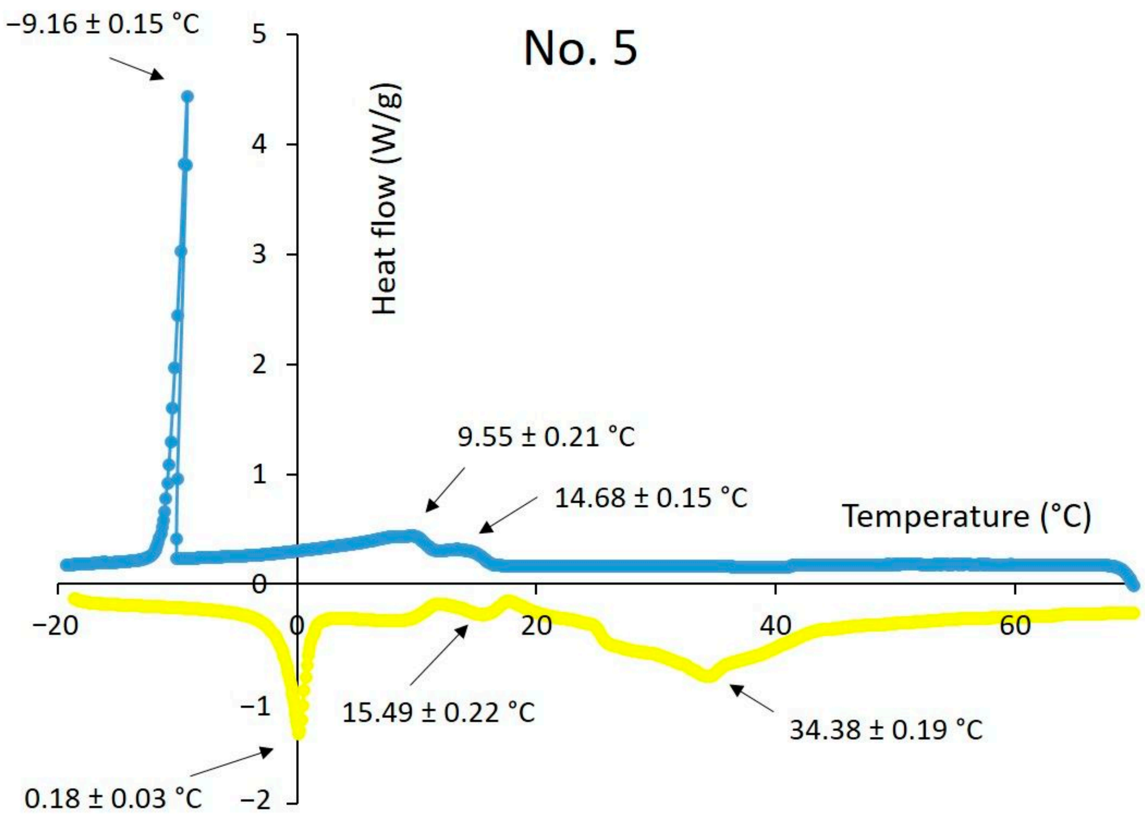

Figure S5: DSC thermograms of detected crystallization peaks on cooling curve (blue colour) and melting peaks on heating curve (yellow colour) for sample No. 5.

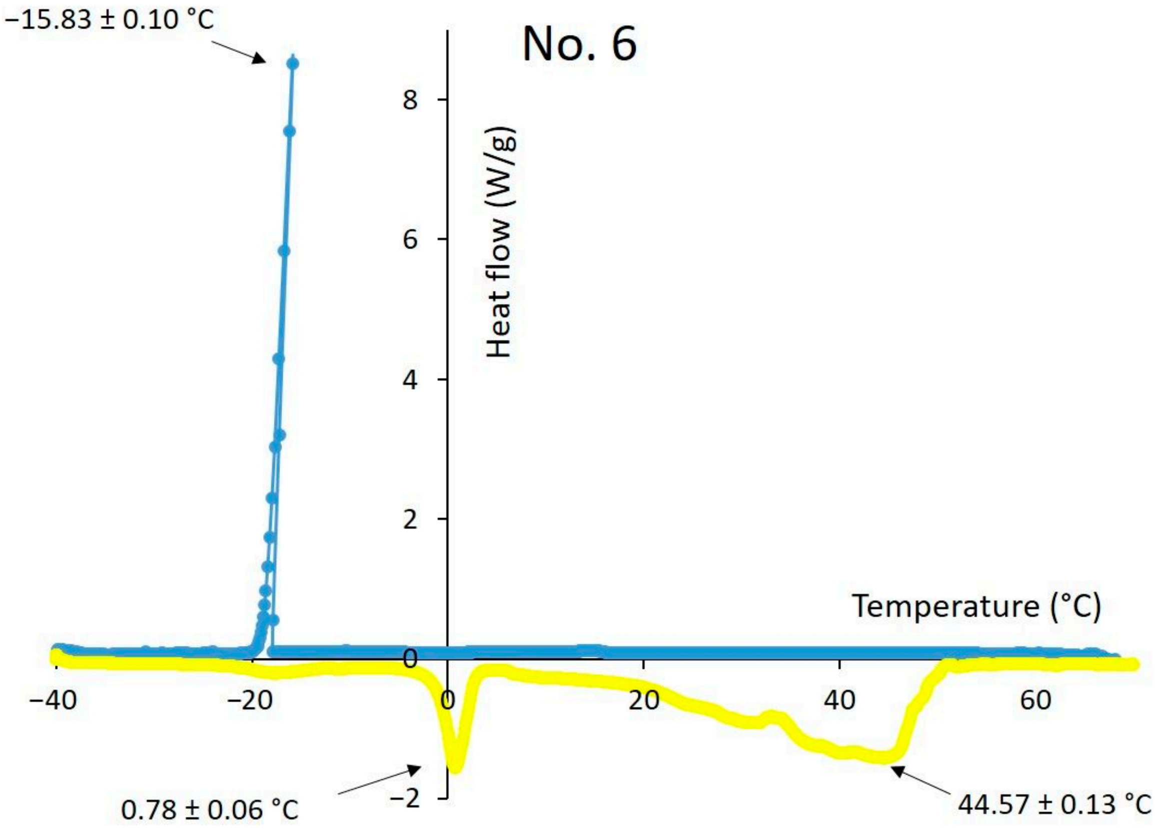

Figure S6: DSC thermograms of detected crystallization peaks on cooling curve (blue colour) and melting peaks on heating curve (yellow colour) for sample No. 6.

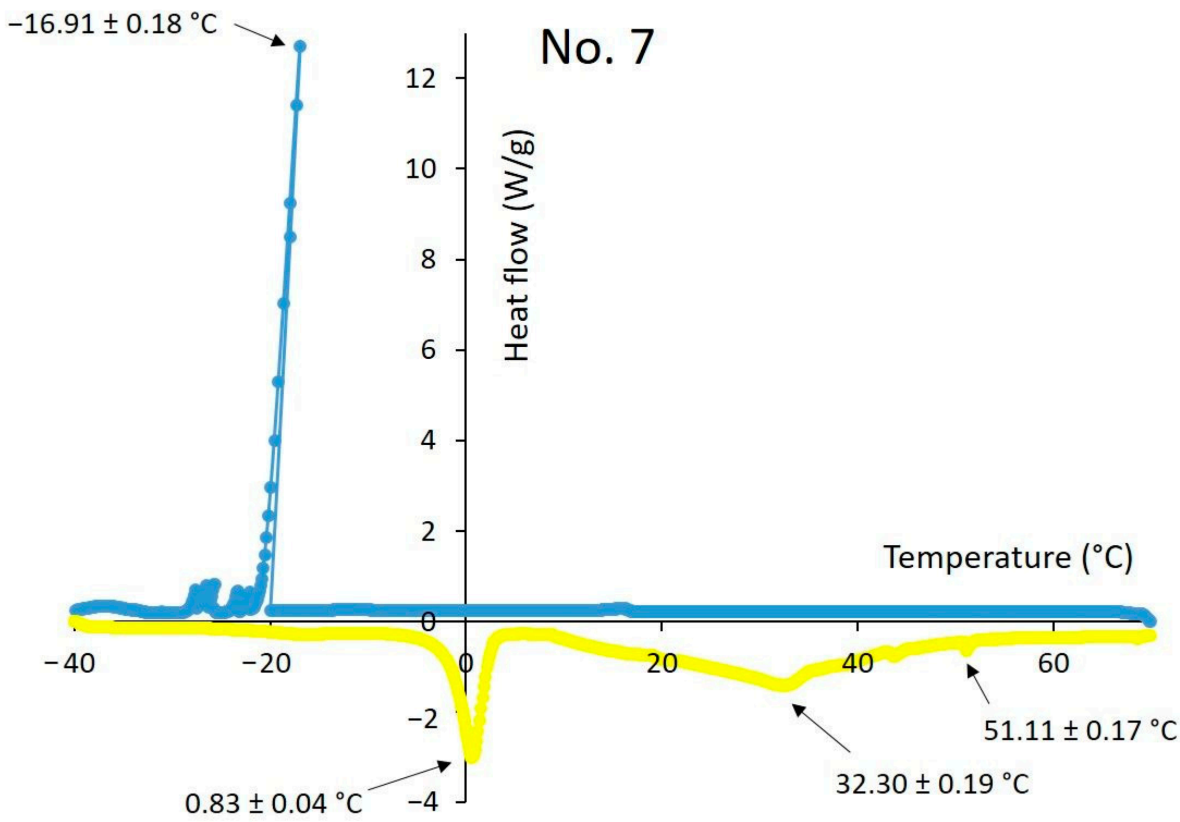

Figure S7: DSC thermograms of detected crystallization peaks on cooling curve (blue colour) and melting peaks on heating curve (yellow colour) for sample No. 7.

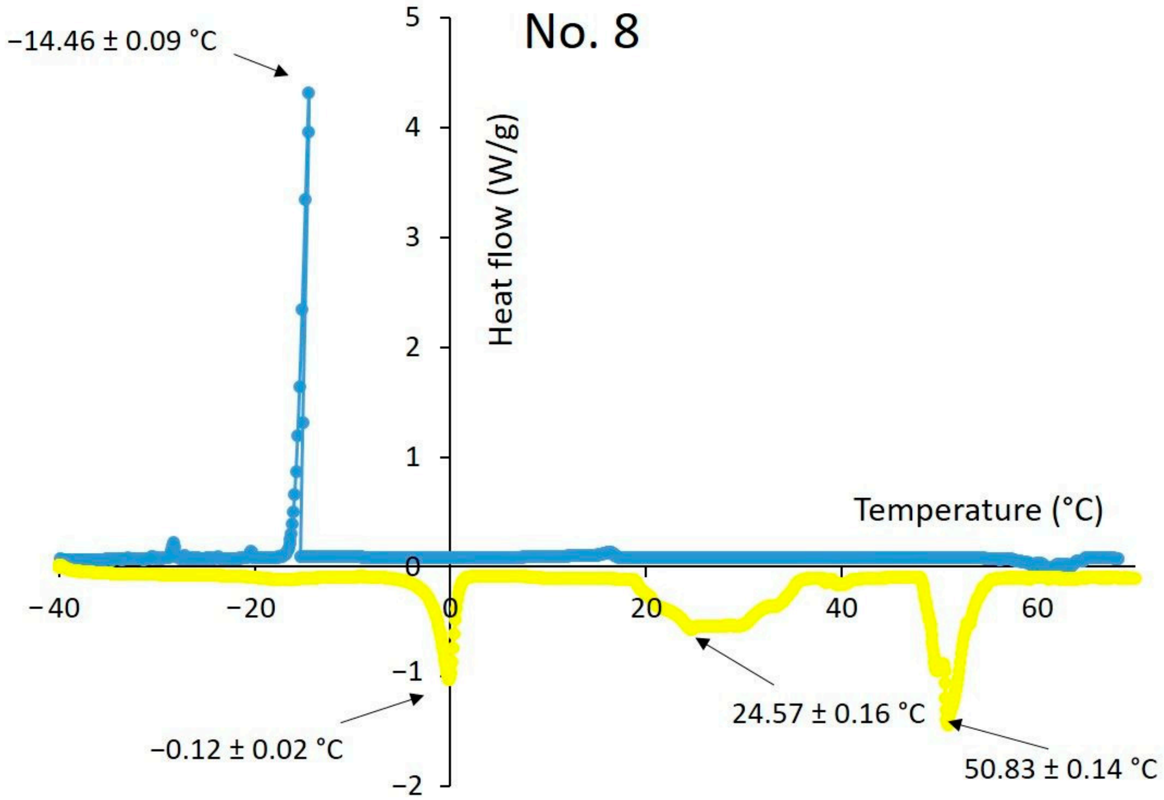

Figure S8: DSC thermograms of detected crystallization peaks on cooling curve (blue colour) and melting peaks on heating curve (yellow colour) for sample No. 8.

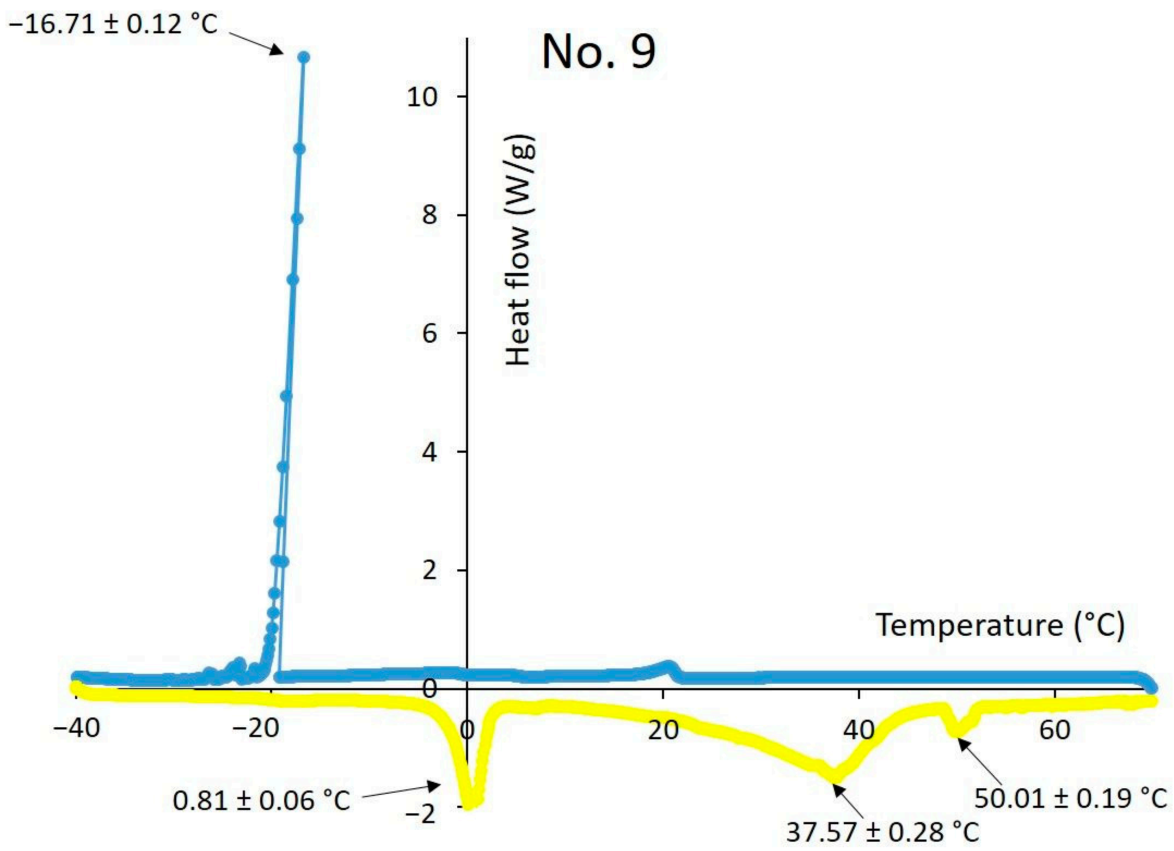

Figure S9: DSC thermograms of detected crystallization peaks on cooling curve (blue colour) and melting peaks on heating curve (yellow colour) for sample No. 9.

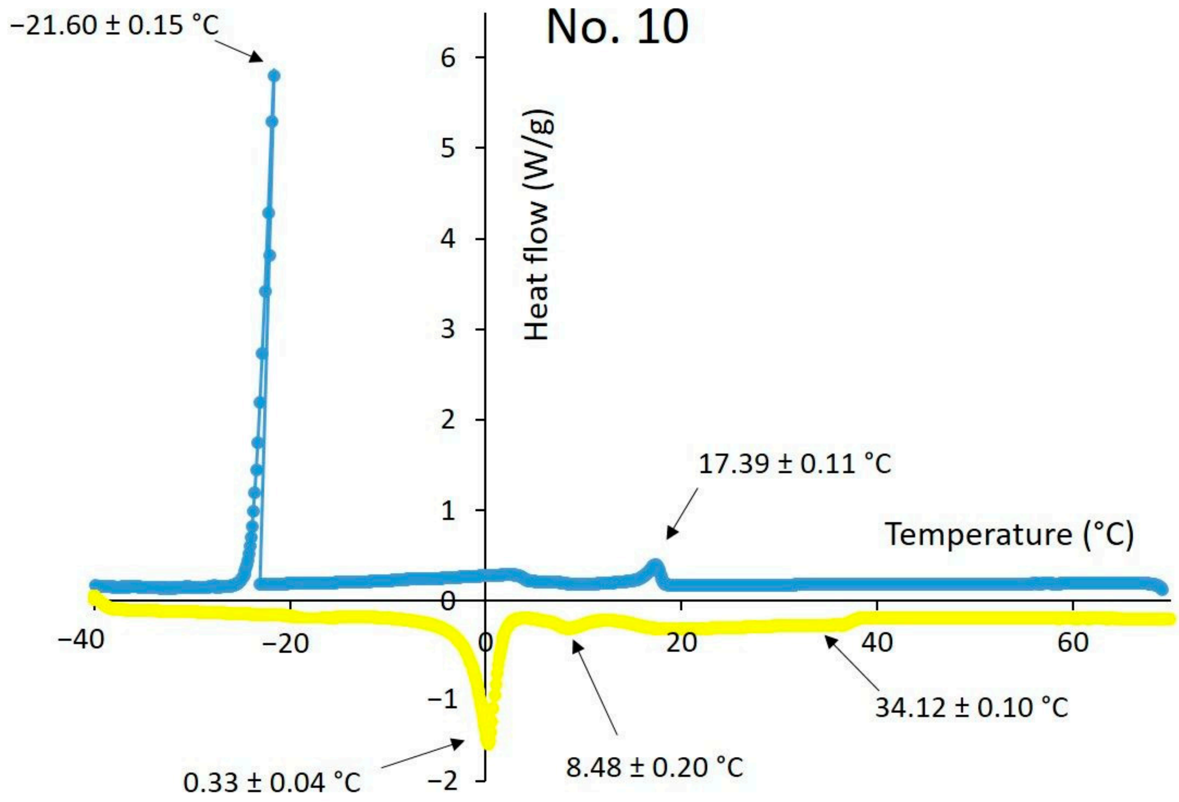

Figure S10: DSC thermograms of detected crystallization peaks on cooling curve (blue colour) and melting peaks on heating curve (yellow colour) for sample No. 10.
